# Efficacy of traditional Chinese medication Tangminling pill in Chinese patients with type 2 diabetes

**DOI:** 10.1042/BSR20181729

**Published:** 2019-04-30

**Authors:** Jing Cheng, Jia Zheng, Yanping Liu, Panpan Hao

**Affiliations:** 1 Key Laboratory of Cardiovascular Remodeling and Function Research, Chinese Ministry of Education and Chinese Ministry of Health, Shandong University Qilu Hospital, Jinan, Shandong, and Shenzhen Research Institute of Shandong University, Shenzhen, Guangdong, China; 2 Department of Endocrinology, Peking University First Hospital, Beijing, China; 3 Department of Radiology, Shandong University Qilu Hospital, Jinan, Shandong, China

**Keywords:** Chinese herbal medication, Diabetes, Tangminling

## Abstract

The morbidity of type 2 diabetes mellitus (T2DM) has been increasing rapidly worldwide. Tangminling pill, consisting of ten Chinese herbal medications, is usually prescribed for T2DM in mainland China. Whether treatment with Tangminling can improve clinical outcomes of T2DM patients was still debated. Four studies comparing Tangminling vs. placebo treatment in T2DM patients were included and 767 T2DM patients were enrolled in our analyses. Tangminling treatment exhibited better efficacy than placebo in reducing hemoglobin A1c (HbA1c) (1.11 vs. 0.32%; pooled weighted mean difference [WMD]: 0.80; 95% confidence interval [CI]: 0.65–0.96; *P*<0.001), fasting plasma glucose (0.82 vs. −0.40 mM; WMD: 1.10; 95% CI: 0.56–1.64; *P*<0.001), 2-h postprandial glucose (2-hr PG) (2.81 vs. 1.11 mM; WMD: 1.80; 95% CI: 1.72–1.88; *P*<0.001), homeostatic model assessment-β level (4.28 vs. 0.41; WMD: 0.44; 95% CI: 0.27–0.61; *P*<0.001), waist circumference (WC) (1.04 vs. 0.36 cm; WMD: 0.78; 95% CI: 0.37–1.19; *P*<0.001) and body weight index (0.37 vs. 0.11 kg/m^2^; WMD: 0.30; 95% CI: −0.00 to 0.61; *P*=0.05). Tangminling pill might reduce glucose level and body weight and improve β-cell function in T2DM patients. Our study highlights the important role of Tangminling pill in the management of T2DM.

## Introduction

Type 2 diabetes mellitus (T2DM) is becoming a crucial threat to human health, with approximately 425 million individuals worldwide in 2017, and it is estimated that the number will exhibit a 45% increase in three decades [1]. T2DM patients might be suffering from pancreatic β-cell dysfunction and reduced insulin sensitivity in target tissues [2,3]. Chronic hyperglycemia causes macrovascular and microvascular complications and remodeling in the heart, kidneys, eyes, and nerves [4–6]. Oral antidiabetic agents, including metformin, sulfonylureas, glucosidase inhibitors, thiazolidinediones, meglitinides, and inhibitors of dipeptidyl peptidase 4, have substantially improved the prognosis of most T2DM patients. However, the efficacy of Western medications remains deficient in certain patients and vastly disproportionate burden on patients in low- and middle-income countries. Hence, complementary or alternative therapies such as traditional Chinese herbal medications are required to prolong life expectancy and improve the quality of life in T2DM patients.

Although traditional Chinese herbal medications have been widely used for more than 2000 years in East Asia and previous studies suggested that some Chinese herbal medications were effective in treating T2DM [7–9], their evidence based on randomized controlled trials (RCTs) is still feeble. Tangminling pill, mainly containing *Rhizoma coptidis, Rheum officinale Baill, Scutellaria baicalensis Georgi*, and *Bupleurum Chinense DC*, might reduce blood glucose level and improve pancreatic β-cell function [8,10–12]. However, the studies focusing on the effects of Tangminling pill on T2DM were limited due to insufficient sample sizes and inconclusive results. In our study, we performed a pooled analysis to evaluate the effects of Tangminling pill on blood glucose, body weight, and β-cell function in T2DM patients.

## Materials and methods

### Preparation of Tangminling pill

The pharmaceutical composition of Tangminling pill comprises extracts of the following ten traditional Chinese herbs: *Rhizoma coptidis* (Huanglian), *Scutellaria baicalensis Georgi* (Huangqin), *Radix Paeoniae Alba* (Baishao), *Rheum officinale Baill* (Dahuang), *Citrus aurantium L* (Zhishi), *Pinellia ternate* (Baixia), *Crataegus pinnatifida Bunge* (Shanzha), *Smoked plum Fructus mume* (Wumei), *Radix Trichosanthis* (Tianhuafen), and *Bupleurum Chinense DC* (Chaihu), and optionally comprises pharmaceutically acceptable excipient. The method to prepare Tangminling pill and the doses of all the above ten crude herbs have been disclosed to the public (http://www.freepatentsonline.com/8637092.html). It was manufactured and capsulated with specific processing of Chinese herbal medicine in Tasly Pharmaceutical (Tianjin, China), as described previously [8]. There were quantitative control limits for the raw herbs in the final product (the content of berberine in *Rhizoma coptidis* shall not be <6.0 mg/g and the content of paeoniflorin in *Radix Paeoniae Alba* shall not be <3.0 mg/g). The chemical composition of Tangminling pill was further analyzed using the high-performance liquid chromatography/mass spectrometry (HPLC/MS) method [8].

### Ethics statement

The study project conformed to the ethical rules of the Helsinki Declaration (revised edition in October 2013) and was approved by the Ethics Committee of Shandong University Qilu Hospital (KYLL-201761; date of approval: 15 July 2017).

### Data search and selection

Relevant RCTs were identified from the following data sources: Medline, China VIP, China National Knowledge Internet, China Wanfang, and Cochrane library. Literature were searched with the terms (‘Tang-min-ling’ or ‘TM81’ or ‘Tangminling’ or ‘TML’) AND (‘diabetes’ or ‘T2DM’). Publication status, language, and date were not restricted.

We included studies with the following criteria: (i) compared Tangminling treatment vs. placebo treatment in T2DM patients; (ii) RCTs with more than 1-month follow-up durations; and (iii) contained available data to estimate the pooled weighted mean differences (WMDs) and their corresponding 95% confidence intervals (CIs). Studies were excluded as follows: (i) diagnosis of T2DM was not definite; (ii) studies were non-randomized and/or non-double-blinded; (iii) different traditional Chinese medications were compared between two groups; (iv) only symptomatic changes were described without objective laboratory measurements; (v) methodological quality was poor with a Jadad score < 3 as described previously [13]; and (vi) if two articles reported results from the same trial, the article with less information was excluded.

### Data extraction

We extracted the following information from retrieved reports using a standardized protocol: author names, publication dates, study designs, countries of origin, subject numbers, subject characteristics at baseline, follow-up durations, concomitant medications, and end points [14]. The literature search and data extraction were performed blindly by two authors (J.C. and Y.L.) according to a standardized approach. All disagreements were resolved by consensus and a third reviewer (P.H.).

### Statistical analysis

RevMan software version 5.3 was used for this pooled analysis. The heterogeneity was evaluated by the Cochran’s Q statistic and the inconsistency index (*I^2^*). Heterogeneity was considered statistically significant when chi-square *P* was <0.10 and *I^2^* was >50%. Pooled WMDs with 95% CIs were analyzed, and the difference was considered statistically significant when two-tailed *P* was <0.05. Moreover, sensitivity analyses were performed to evaluate individual effects and explain possible heterogeneity. Additionally, we calculated the fail-safe number (Nfs; N_fs0.05_ = (∑Z/1.64)^2^ – k, where k is the number of retrieved studies) to assess publication bias [15]. Any N_fs0.05_ values smaller than the number of included trials indicated that obvious publication bias existed.

## Results

Two hundred and seventy eight potentially literature citations were screened. Only four RCTs with sufficient information were included for analyses, and all the four RCTs were published in full articles updated to December 2017 [8,10–12]. The agreement for literature selection was excellent (κ = 0.93). Seven hundred and sixty seven T2DM patients were enrolled in our pooled analyses, with 71–397 participants in the four eligible trials. The mean ages of the participants ranged from 51.6 to 54.5 years, and the follow-up durations were all 12 weeks. The Jadad scores were five in three of four RCTs [10–12] and four in the left one RCT [8]. The loss to follow-up was <10% in all RCTs. Thus, the methodological quality of the retrieved studies was generally high.

### Clinical end points

First, we performed a pooled analysis to evaluate the effect of Tangminling pill on blood glucose level. Blood glucose improvement was evaluated by reductions of hemoglobin A1c (HbA1c), fasting plasma glucose (FPG) and 2-h postprandial glucose (2-hr PG) levels from baseline in all four studies (Table 1). It showed that Tangminling pill significantly reduced HbA1c (1.11 vs. 0.32%, *P*<0.001), FPG (0.82 vs. −0.40 mM, *P*<0.001) and 2-hr PG levels (2.81 vs. 1.11 mM, *P*<0.001) in T2DM patients, as compared with placebo group.

**Table 1:** Main results of clinical outcomes

Outcomes	Placebo-treated patients, *n*	Tangminling pill-treated patients, *n*	WMD (95% CI)	*P-*value	*I*^2^ statistic	Heterogeneity *P-*value	References
HbA1c	259	440	0.80 [0.65, 0.96]	<0.001	0%	>0.05	[8,10–12]
FPG	259	440	1.10 [0.56, 1.64]	<0.001	77%	<0.01	[8,10–12]
2-hr PG	259	440	1.80 [1,72, 1.88]	<0.001	0%	>0.05	[8,10–12]
BMI	259	440	0.30 [-0.00, 0.61]	0. 05	90%	0.001	[8,10–12]
WC	259	440	0.78 [0.37, 1.19]	<0.001	63%	<0.05	[8,10–12]
HOMA-IR	190	373	0.26 [-0.10, 0.62]	>0.05	81%	<0.01	[8,10,12]
HOMA-β	190	494	0.44 [0.27, 0.61]	<0.001	36%	>0.05	[8,10,12]

Abbreviations: BMI, body weight index; HOMA-β, homeostatic model assessment to β-cell function; HOMA-IR, homeostatic model assessment to quantitate insulin resistance; PG, postprandial plasma glucose; WC, waist circumference.

Also, Tangminling pill significantly ameliorated obesity, which was expressed as reductions of waist circumference (WC) and body mass index (BMI) in T2DM patients (Table 1). Tangminling significantly reduced WC (1.04 vs. 0.36 cm, *P*<0.001) and BMI (0.37 vs. 0.11 kg/m^2^, *P*=0.05) from baseline in comparison with placebo treatment in T2DM patients.

Furthermore, it showed that Tangminling pill significantly improved pancreatic β-cell function, expressed as homeostatic model assessment (HOMA)-β decrease from baseline (Table 1). Although there was no difference in reducing HOMA-insulin resistance (IR) between Tangminling and placebo groups (0.16 vs. −0.16, *P*>0.05), Tangminling significantly decreased HOMA-β level from baseline as compared with placebo (4.28 vs. 0.41, *P*<0.001).

Furthermore, the safety of Tangminling pill was also confirmed, without serious adverse events reported in all the four RCTs.

### Sensitivity analyses

Heterogeneity was well-addressed in terms of HbA1c (*I^2^* = 0%), 2h-PG (*I^2^* = 0%), and HOMA-β (*I^2^* = 36%). However, significant heterogeneity was existed in terms of FPG (*I^2^* = 77%), BMI (*I^2^* = 90%), WC (*I^2^* = 63%), and HOMA-IR (*I^2^* = 81%). Alterations in pooled WMDs were not significant after excluding the study with the largest sample size [8] (data not shown). Thereafter, subgroup analyses according to FPG, BMI, WC, and HOMA-IR were conducted (Table 2). The heterogeneity among trials was partially explained by the variability in doses of the medication and subject numbers.

**Table 2:** Subgroup analyses concerning FPG, BMI, WC, and HOMA-IR

Outcome	Subgroup	Patients, *n*	WMD (95% CI)	*P-*value	*I^2^* statistic	Heterogeneity *P*-value	References
**FPG**	**Adjustment for doses**						
	12 g	208	0.99 [0.37, 1.62]	<0.01	13%	>0.05	[11,12]
	6 g	628	1.22 [0.39, 2.04]	<0.01	86%	<0.001	[8,10,11]
	**Adjustment for subject numbers**						
	>100	672	1.01 [0.41, 1.61]	0.001	77%	0.01	[8,11]
	≤100	164	1.66 [1.04, 2.28]	<0.001	0%	>0.05	[10,12]
**BMI**	**Adjustment for doses**						
	12 g	208	0.21 [−0.31, 0.73]	>0.05	0%	>0.05	[11,12]
	6 g	628	0.33 [−0.33, 0.69]	>0.05	95%	<0.001	[8,10,11]
	**Adjustment for subject numbers**						
	>100	672	0.53 [0.36, 0.70]	<0.001	13%	>0.05	[8,11]
	≤100	164	0.16 [0.03, 0.29]	0.01	0%	>0.05	[10,12]
**WC**	**Adjustment for doses**						
	12 g	208	0.57 [0.06, 1.08]	<0.05	0%	>0.05	[11,12]
	6 g	628	0.84 [0.32, 1.36]	<0.01	70%	0.04	[8,10,11]
	**Adjustment for subject numbers**						
	>100	672	1.10 [1.08, 1.12]	<0.001	0%	>0.05	[8,11]
	≤100	164	0.53 [0.19, 0.88]	<0.01	0%	>0.05	[10,12]
**HOMA-IR**	**Adjustment for doses**						
	12 g	71	0.68 [0.19, 1.17]	<0.01	NA	NA	[11,12]
	6 g	492	0.10 [−0.17, 0.38]	>0.05	71%	>0.05	[8,10,11]
	**Adjustment for subject numbers**						
	>100	399	0.00 [−0.02, 0.02]	>0.05	NA	NA	[8]
	≤100	164	0.40 [0.14, 0.66]	<0.01	43%	>0.05	[10,12]

### Publication bias

N_fs0.05_ values for HbA1c, FPG, 2-hr PG, BMI, WC, HOMA-β, and HOMA-IR were higher than the number of studies included in the corresponding analyses, indicating negligible publication bias in our analyses.

## Discussion

The pooled analyses indicated that Tangminling pill was more efficacious in decreasing glucose level, body weight, and improving β-cell function, with no more drug-related adverse events, than placebo treatment in T2DM patients.

Traditional Chinese medications, mainly edible herbs, have been widely used in clinical practice in China for more than 2000 years [16–18]. Ten traditional Chinese herbs were extracted and included in Tangminling pill: *Rhizoma coptidis, Scutellaria baicalensis Georgi, Radix Paeoniae Alba, Rheum officinale Baill, Citrus aurantium L, Pinellia ternate, Crataegus pinnatifida Bunge, Smoked plum Fructus mume, Radix Trichosanthis* and *Bupleurum Chinense DC* (Table 3). Three bioactive alkaloids have been isolated from *Rhizoma coptidis*, including berberine, coptisine, and jatrorrhizine. Berberine has numerous pharmacological effects with anti-diabetic, anti-inflammatory, anti-hypercholesterolemia, and cardioprotective properties [19–23]. Berberine displays beneficial actions in the treatment of insulin-resistant and hyperglycemic states by up-regulating adipose triglyceride lipase in adipocytes via activating adenosine 5′-monophosphate (AMP)-activated protein kinase (AMPK) signaling pathway [24,25]. It might improve glucose metabolism via inhibiting oxidation and aldose reductase. Besides, berberine might exert its anti-inflammatory action through AMPK activation, nuclear factor-κB (NF-κB) inhibition, and AP-1 pathway inhibition [26,27]. Coptisine is another bioactive component of *Rhizoma coptidis*, and it exhibits antioxidation, anti-osteosarcoma, anti-inflammation, neuroprotection, and anti-cancer actions through preventing interleukin-1β (IL-1β) expression, activating Akt and c-Jun N-terminal kinase (JNK)/nuclear factor erythroid 2-related factor 2 (Nrf2)/NAD(P)H:quinine oxidoreductase 1 (NQO1) signaling pathways and inhibiting lipopolysaccharide (LPS)/toll-like receptor-4 (TLR-4)-mediated pathway [28–32]. *Rheum officinale Baill*, the root and rhizome of *Rheum palmatum L*, has antioxidant, anti-platelet aggregation, and anti-inflammatory properties [33–35]. The underlying mechanism might involve reductions in the mRNA expressions of acidic mammalian chitinase and chitinase 3-like protein 4 as well as inhibition of the progression of inflammation [35]. *Bupleurum Chinense DC* and its bioactive ingredients, saikosaponins, exhibited immunomodulatory [36], antiviral [37], hepatoprotective [38], and anti-cancer [39] effects through inducing cell cycle arrest and cell apoptosis [40]. Moreover, albiflorin, which was extracted from *Radix Paeoniae Alba* or *Paeonia lactiflora*, exerted anti-depressant and anti-allergic effects through biosynthesis of allopregnanolone and inhibition of mast cell activation [41,42]. The main active ingredients of *Scutellaria baicalensis Georgi* are baicalin and wogonin, which exhibited protection of diabetes and its complications. Baicalin reduced renal fibrosis through down-regulating transforming growth factor-β/Smad pathway [43]. It also had a protective effect on T2DM via improving islet β-cell function [44]. Furthermore, wogonin alleviated diabetic cardiomyopathy, diabetes-related cognitive deficits, and metabolic syndrome [45–47]. Wogonin improved peroxisome proliferator-activated receptor (PPAR)-α (PPAR-α) activity in adipocytes and type 1 diabetic mice [48]. Flavanones naringenin and hesperetin from *Citrus aurantium L* exhibited anti-depressant [49], antioxidant [50], and anti-atherogenic [51] effects. *Pinellia ternate* had anti-cancer [52,53] and pro-inflammatory [54] actions. It also attenuated mucus secretion and airway inflammation in rats with chronic obstructive pulmonary disease [55]. *Crataegus pinnatifida Bunge* exerted anti-inflammatory effects [56] and decreased the risks of cardiovascular diseases by lowering blood cholesterol [57–59]. *Fructus mume* exhibited neuroprotective effect through down-regulating inflammation and inhibiting TLR4 and p38 MAPK signaling [60,61]. *Radix Trichosanthis* played anti-cancer [62] and immunosuppressive [63] roles. The combination of these components in Tangminling pill might exert more beneficial effects in T2DM patients. In our study, the safety of Tangminling pill was also confirmed, without serious adverse events reported in the included RCTs. However, the production of traditional Chinese medicine is very complicated, so it is necessary to test the effectiveness and safety of each plant extract and investigate easier preparation methods of the pill formulation.

**Table 3:** Major herbs and ingredients of Tangminling pill and potential mechanisms

Herbs	Bioactive ingredients	Beneficial effects	Potential mechanisms	Experimental models used
***Rhizoma coptidis***	Berberine	- anti-inflammatory [23, 24]; - anti-hypercholesterolemia [21, 22]; blood glucose-lowering [20]; - anti-obesity [21]; - cardioprotective [19]; and antioxidative [17]	- inhibiting PPAR-γ and C/EBP-α [21]; increasing adipose triglyceride lipase [20]; - inhibiting NF-κB [23, 24]; AMPK activation [22–24]	Cell cultures; rodent models
**Rhizoma coptidis**	Coptisine	- antioxidative [25]; - anti-inflammatory [26]; - neuroprotective [27]; - anti-obesity [28]; and anti-cancer [29]	- preventing IL-1β expression; activating Akt and JNK/Nrf2/NQO1 pathway [25]; - inhibiting LPS/TLR-4-mediated signaling pathway [28]	Cell cultures; rodent models
**Rheum officinale Baill**	Rhubarb	- antioxidative [30]; - anti-platelet aggregation [31] and anti-inflammatory [32]	- regulating inflammatory mediators [32]	Cell cultures; rodent models
**Bupleurum Chinense DC**	Saikosaponins	- immunomodulatory [33]; antiviral [34]; - hepatoprotective [35]; - anti-cancer [36]	- cell cycle arrest and induction of apoptosis [37]	Cell cultures; rodent models
**Radix Paeoniae Alba**	Albiflorin	- antidepressant [38] and anti-allergic [39]	- allopregnanolone biosynthesis; - inhibition of mast cell activation [39]	Cell cultures; rodent models
**Scutellaria baicalensis Georg**	Baicalin	- anti-fibrosis [40]; lipid-lowing; anti-hyperglycemic [41]; - alleviating diabetes-associated cognitive deficits [44]	- modulating TGF-β/Smad signaling pathway [40]; - promoting islet β-cell function [41]; - modulation of MAPK sighnaling [44]	Cell cultures; rodent models
**Scutellaria baicalensis Georg**	Wogonin	- cardioprotective [42]; - anti-inflammatory [42]; - antioxidative [42]; - anti-hyperglycemic and lipid-lowering [43]	- activating PPARα [43, 45]; - decreasing ROS [42];	Cell cultures; rodent models
**Citrus aurantium L**	Naringenin/hesperetin	- antioxidant [47]; - antiatherogenic [48] and antidepressant [46]	- increasing expression of BDNF [46]; - activating PPAR; - up-regulating adiponectin expression [48]	Cell cultures; rodent models
**Pinellia ternata**	Pinellia ternata lectin	- pro-inflammatory [51]; - anti-cancer [49]; anti-tumor [50]; - increasing steroid biosynthesis [52]	- inhibits cell proliferation and metastasis [49]; - inhibiting ERK activation [52] and activating NF-κB [51]	Cell cultures; rodent models
**Crataegus pinnatifida Bunge**	Haw pectin pentasaccharide	- anti-inflammatory [53]; - antioxidant [55]; - lowering blood cholesterol [56] and anti-atherogenic [54]	- activating PPARα and acyl-CoA oxidase [55]	Cell cultures; rodent models
**Fructus mume**	Fructus mume extract	- alleviating cognitive impairments [57] and anti-inflammatory [58]	- down-regulation of TLR4 and p38 MAPK signaling [58]	Cell cultures; rodent models
**Radix Trichosanthis**	Trichosanthin	- anti-tumor [59]; anti-HIV; immunosuppressive [60]	- inhibiting cell growth and inducing apoptosis [59]	Cell cultures; rodent models

Abbreviations: BDNF, brain derived neurophic factor; C/EBP-α, CCAAT enhancer binding protein-α; ERK, extracellular signal-regulated kinase; JNK, c-Jun N-terminal kinase; MAPK, mitogen-activated protein kinase; ROS, reactive oxygen species; TGF-β, transforming growth factor-β.

T2DM is a chronic metabolic disease in which the deterioration of glycemic control is associated with progressive islet β-cell dysfunction, chronic inflammation, oxidation, and lipid metabolic disturbance. Metabolic syndrome is characterized by the presence of abdominal obesity, IR, hyperglycemia, dyslipidemia, and hypertension [64]. Tangminling pill significantly improved pancreatic β-cell function and reduced BMI and WC in T2DM patients in our analyses. The anti-hyperglycemic effect of traditional Chinese medications might be attributed in part to the β-cell protection through activating AMPK signaling [65]. Tangminling was found to increase glucose transporter 4 (GLUT4) level and AMPK activity in skeletal muscle [66] and promote insulin secretion through up-regulating PPARα [67]. It might also promote the proliferation and differentiation of pancreatic β-cells and therefore postpone the exhaustion of β-cell function. Tangminling was showed to improve insulin sensitivity [68], which might be another potential mechanism to regulate glucose metabolic disorder.

Hypoglycemia has been considered as a major barrier to the proper glycemic control in diabetic patients [69]. Our analyses revealed that Tangminling reduced blood glucose mildly, without hypoglycemic events reported. Tangminling treatment might significantly decrease the risk of hypoglycemia, which result in a better quality of life for T2DM patients. Besides, significant heterogeneity existed in the analyses of FPG, BMI, WC, and HOMA-IR. There was a significant disparity in sample sizes among the four RCTs included in our pooled analyses. In fact, among the 767 patients included in the analyses, 399 (52.0%) were from one study [8]. However, after excluding this study, no substantial alteration was found in pooled WMDs.

Several limitations should be considered in our analyses. The sample sizes were relatively small, and the number of included studies was limited, both of which might limit the power to estimate the effects of Tangminling pill on T2DM. Intervention and follow-up durations of some studies were relatively short. Therefore, more high-quality studies with more extended intervention and larger sample sizes are needed. Despite the above limitations, this systematic analysis was more convincing than any previous single study. Indeed, our analysis has some notable strengths. It strictly adhered to the guidelines and only RCT studies were included, which helps to increase the robustness of the conclusions. In addition, the parameters used to assess the clinical outcomes are strict, which can increase the validity of our study. To the best of our knowledge, this is the first pooled analysis to assess the effect of one traditional Chinese medication, Tangminling pill, on T2DM.

## Conclusion

Tangminling pill is effective and safe for glycemic control and β-cell function in T2DM patients, and it could be used to treat T2DM as a promising complementary approach. However, more rigorously designed trials are warranted to investigate the impacts of Tangminling pill on diabetes and diabetic complications.

## Abbreviations

AMPK: AMP-activated protein kinase
BMI: body mass index
CI: confidence interval
FPG: fasting plasma glucose
HbA1c: hemoglobin A1c
HOMA: homeostatic model assessment
IR: insulin resistance
*I^2^*: inconsistency index
Nfs: fail-safe number
PPAR: peroxisome proliferator-activated receptor
RCT: randomized controlled trial
T2DM: type 2 diabetes mellitus
WC: waist circumference
WMD: weighted mean difference
2-hr PG: 2-h postprandial glucose
